# Using individual barcodes to increase quantification power of massively parallel reporter assays

**DOI:** 10.1186/s12859-025-06065-9

**Published:** 2025-02-13

**Authors:** Pia Keukeleire, Jonathan D. Rosen, Angelina Göbel-Knapp, Kilian Salomon, Max Schubach, Martin Kircher

**Affiliations:** 1 Institute of Human Genetics, University Hospital Schleswig-Holstein, University of Lübeck, Lübeck, Germany; 2 Department of Genetics & Department of Biostatistics, University of North Carolina at Chapel Hill, Chapel Hill, NC USA; 3 Exploratory Diagnostic Sciences, Berlin Institute of Health at Charité – Universitätsmedizin Berlin, Berlin, Germany

**Keywords:** Massively Parallel Reporter Assays (MPRA), Element and variant effect quantification, Statistical analysis, R package

## Abstract

**Background:**

Massively parallel reporter assays (MPRAs) are an experimental technology for measuring the activity of thousands of candidate regulatory sequences or their variants in parallel, where the activity of individual sequences is measured from pools of sequence-tagged reporter genes. Activity is derived from the ratio of transcribed RNA to input DNA counts of associated tag sequences in each reporter construct, so-called barcodes. Recently, tools specifically designed to analyze MPRA data were developed that attempt to model the count data, accounting for its inherent variation. Of these tools, MPRAnalyze and mpralm are most widely used. MPRAnalyze models barcode counts to estimate the transcription rate of each sequence. While it has increased statistical power and robustness against outliers compared to mpralm, it is slow and has a high false discovery rate. Mpralm, a tool built on the R package Limma, estimates log fold-changes between different sequences. As opposed to MPRAnalyze, it is fast and has a low false discovery rate but is susceptible to outliers and has less statistical power.

**Results:**

We propose BCalm, an MPRA analysis framework aimed at addressing the limitations of the existing tools. BCalm is an adaptation of mpralm, but models individual barcode counts instead of aggregating counts per sequence. Leaving out the aggregation step increases statistical power and improves robustness to outliers, while being fast and precise. We show the improved performance over existing methods on both simulated MPRA data and a lentiviral MPRA library of 166,508 target sequences, including 82,258 allelic variants. Further, BCalm adds functionality beyond the existing mpralm package, such as preparing count input files from MPRAsnakeflow, as well as an option to test for sequences with enhancing or repressing activity. Its built-in plotting functionalities allow for easy interpretation of the results.

**Conclusions:**

With BCalm, we provide a new tool for analyzing MPRA data which is robust and accurate on real MPRA datasets. The package is available at https://github.com/kircherlab/BCalm.

**Supplementary Information:**

The online version contains supplementary material available at 10.1186/s12859-025-06065-9.

## Background

A massively parallel reporter assay (MPRA) is a type of experimental assay that allows for parallel testing of activating and repressing expression effects of thousands of gene regulatory sequences on a reporter construct in a single experiment. In a typical MPRA experiment, tested sequences are associated with multiple short sequence tags, so-called barcodes. The regulatory sequences with their respective barcodes are cloned into a plasmid with a reporter gene (typically a luciferase or fluorescent protein), with the barcodes being expressed as part of the 5’ or 3’ untranslated regions (UTRs) of the reporter gene transcripts and the tested sequence being located directly upstream of the transcriptional start site (TSS) or a minimal (or otherwise selected) promoter sequence. The plasmid libraries can be transfected into different cell populations and expressed episomally, or integrated into the genomic sequence, for example using a lentiviral vector system (lentiMPRA). With stable expression of the plasmid constructs in the cell-types of interest, sequencing the barcodes from transcribed RNA and the plasmid DNA allows for quantifying the activity of the sequence via the barcode [1–3]. By testing multiple alternative sequences, e.g. by creating individual allelic substitutions or small insertion/deletion changes, the effects of sequence variants can be quantified as the activity differences to a selected reference allele [4].

The barcode counts obtained from RNA and DNA extractions are a noisy representation of the underlying activity. They are not just affected by sequencing depth, but also altered by various biological and experimental processes. The counts are prone to overdispersion, meaning there is greater variability than expected based on typical count models like the Poisson distribution [5]. Additionally, there are biases originating from different technical replicates [6], and barcodes associated with the same sequence have varying activity levels due to their sequence properties. For example, some barcodes show abnormal high activity due to certain base compositions (e.g., high or low Guanine and Cytosine (GC) content) or their creation of RNA binding protein (RBP) and microRNA target binding sites [7]. In lentiMPRA, the location of the construct integration site in the genome (e.g., in open or closed chromatin) has an effect on the activity as well [8–10]. To accurately quantify and analyze the effects of the tested sequences, it is crucial to have multiple barcodes tagging the same tested sequence, to ensure that numerous cells carry (DNA) and express (RNA) each tagged reporter construct, and to account for the noise and variation inherent in MPRA count data [11].

Commonly, MPRA activity is quantified as the logarithm to the base 2 (log2) of the RNA over the DNA counts, herein referred to as logratio. The resulting logratios are sometimes analyzed using software originally designed for RNA-seq analysis, e.g. edgeR [12] or DESeq2 [13]. However, as opposed to expression data, the plasmid abundance of each sequence cannot be assumed constant in MPRA data, requiring a different approach to correcting for barcode abundance in DNA. To this end, multiple MPRA-specific analysis methods have been designed. Some of these MPRA analysis methods model the RNA and DNA counts separately [6, 14–16], instead of modeling the logratios. While most methods summarize barcode counts per sequence by taking an average, a median, or by summation, there have also been attempts to model the counts of individual barcodes [15, 16]. By modeling the barcode counts individually, the statistical power to detect small effect sizes between different conditions increases while the individual impact of outlier counts decreases [17, 18].

Among all existing methods for analyzing MPRA data [6, 14–16, 19, 20], mpralm [19] and MPRAnalyze [16] are widely used in the field. Both are published R packages suitable for testing both variant effects (the effect of an alternative allele compared to its reference allele) and element effects (the effect of a single sequence compared to a group of baseline control sequences). One difference between the two methods is that mpralm aggregates barcode counts, whereas MPRAnalyze models the individual counts. As a result, MPRAnalyze has increased robustness to outliers and increased statistical power compared to mpralm. While the probability of a Type II error (i.e., false negative) is decreased, we show that it has a higher probability of a Type I error (i.e., false positive), causing a higher false discovery rate (FDR). Another notable difference between the two methods is their scalability to large datasets. While mpralm models a dataset of 5000 variants in less than a minute, MPRAnalyze requires multiple hours. Since currently tested libraries contain tens to hundreds of thousands of sequences [21–23], scalability is a major concern. Hence, mpralm has a low FDR and short run times, while MPRAnalyze is more robust to outliers and has improved statistical power.

In this work, we extend mpralm to use individual barcodes as an input to increase statistical power and outlier robustness, while remaining scalable for large numbers of variants, effectively combining the strengths of mpralm and MPRAnalyze. With the new R software package, BCalm (for BarCode analysis using linear models), we provide the necessary preprocessing code for generating the correct input format directly from MPRAsnakeflow [24], the standard MPRA pipeline of the Impact of Genomic Variation on Function (IGVF) Consortium [25] and the successor of MPRAflow [1], which generates MPRA count tables from sequencing files. Based on the user's specifications, BCalm models either individual barcode counts or aggregated counts to measure either variant or element effects, and provides built-in support for testing element effects.

## Materials and methods

### Data collection

To exemplify the effects of modeling MPRA data, we use a lentiMPRA [1] data set created in HepG2 cells (cell line of epithelial-like morphology isolated from a hepatocellular carcinoma of a 15-year-old) with three technical replicates (IGVF accession identifier: IGVFSM9009DVDG). Sequences tested in this experiment aim at capturing variant effects across tens of thousands of candidate cis-regulatory element (cCRE) sequences of 200 base pair (bp) length (flanked from both sides with a common 15 bp adapter sequence). The cCREs were selected from ENCODE DNase peak calls across multiple cell-lines, and only elements located within 650–20,000 base pairs upstream of gene transcriptional start sites (TSSs) were included. The design excluded those elements with an overlap to TSSs and prioritized variants within the cCREs using a convolutional neural net (CNN) architecture trained across multiple cell-types [26]. Using the CNN model predictions, 120,000 variants with fixed proportions of high, low, or neutral predictive effects were sampled. Additionally, the design included 6,700 (positive and negative) controls, a subset of which is being used for identifying significant expression increasing effects. The assignment and extracted DNA and RNA sequencing data was processed using MPRAsnakeflow [24].

### Variant annotation

To investigate the potential biological mechanisms of variants that are called significant, we ran all variants that are in the significance sets of mpralm and BCalm through Combined Annotation Dependent Depletion (CADD) v1.7 [26] to obtain PHRED-scaled deleterious scores and their underlying annotations. We used the Ensembl Variant Effect Predictor web tool (Ensembl release 112) [27] to find variants that are located within a transcription factor binding site.

### Data simulation

Because of the lack of a ground truth data set for quantitative gene regulation, we simulated MPRA count data for 5000 variants. For each variant the reference count data was drawn from a negative binomial distribution to account for overdispersion [28], where the parameters of each variant’s distribution were derived using a random variant from our lentiMPRA library. For each replicate in the experimental library, we generated two sets of counts, to obtain a total of six simulated replicates. The variants are equally divided into five effect groups: strongly repressing, weakly repressing, neutral, weakly activating and strongly activating. To simulate these effects, we created variants with log2 fold changes (logFCs) of − 2, − 0.5, 0, 0.5, 2. While DNA counts of reference and alternative alleles are identically distributed, the RNA count data for the alternative allele was drawn from a distribution centered at the mean of the reference allele multiplied by a factor of 0.25, $\frac{1}{\sqrt{2}}$, 1, $\sqrt{2}$, and 4 for each of the respective effect groups.

Using the barcode counts empirical mean ($\upmu$) and empirical variance ($\upsigma$) for a single sequence in our lentiMPRA library, $N$ new counts $x$ (either RNA or DNA) for a simulated variant are drawn as follows, where $N$ is the number of barcodes associated to the original sequence:

$$n = \frac{{\mu^{2} }}{{\sigma^{2} - \mu }},\;p = \frac{n}{n + \mu }$$

$${\text{x}}_{{\text{i}}} \sim {\text{NB}}\left( {n,p} \right)\;{\text{for}}\;i = \left\{ {1, \ldots ,N} \right\}$$

Since each real sequence in the lentiMPRA library is associated with a random number of barcodes, by using the same number of barcodes for the simulated variants, we ensure that the distribution of barcodes per sequence in the simulated data matches that of the real data.

### Outlier simulations

For the simulated dataset to better resemble real MPRA libraries, we added additional random barcodes to create outlier counts. Defining an outlier barcode count as any RNA count that is further than three standard deviations away from the mean, a fraction of 0.028 of the barcodes in our actual lentiMPRA library contains an outlier count. Of these outliers, only a small fraction (0.02) has an outlier count in each of the three replicates.

As the actual fraction of outliers can vary in different MPRA datasets, we added outliers at fractions of 0.1, 0.05, 0.01, 0.005, and 0.001 of all barcodes to the simulated dataset. Of these barcodes, one or more replicates of the RNA counts were picked and its count multiplied by 25. The probabilities of creating outliers from 1, 2, 3, 4, 5, or 6 replicates were set to 0.85, 0.05, 0.04, 0.03, 0.02, 0.01, respectively. This follows the observation in the data that most outlier counts occur in only a single replicate. For each fraction, the random addition of outliers was performed five times.

### Data preprocessing

The count tables obtained after running MPRAsnakeflow are filtered for barcodes that contain a DNA and RNA count of at least one in each replicate, and for sequences that are associated with at least 10 barcodes. We analyzed the dataset both before and after a manual outlier removal step. To remove outliers, we remove all barcodes with an RNA count more than three standard deviations away from the mean for that sequence. The BCalm R package provides the function create_var_df to combine count tables with a table containing variant annotations. This variant table should contain the variant ID in one column, and the sequence names of the reference and alternative alleles in two other columns. As the number of columns in the input scales linearly with the number of barcodes per sequence, an optional down sampling step can be performed to limit the maximum number of barcodes per sequence. The downsample_barcodes method determines an upper limit for the number of barcodes used per sequence by calculating the 95th percentile of barcode counts across all sequences. For sequences that have more barcodes than this threshold, it randomly selects a subset of barcodes. Since there are few sequences with an extremely large number of barcodes in our data, we perform this down sampling step on our entire dataset. Next, the DNA and RNA count data is separated to fit to the expected input format of BCalm, mpralm and MPRAnalyze. This is done using create_dna_df and create_rna_df. The resulting data frames can be used to create an MPRASet as input to BCalm. Additionally, BCalm allows the addition of labels to the MPRASet, which are useful when comparing the logratios between sequences of different groups.

The final variant dataset contained 83,336 variants before outlier removal, and 82,258 after. The final element dataset, after outlier removal, contains 166,508 sequences.

### Algorithm

BCalm is an adaptation of mpralm (version 1.26.2) [19], which in turn utilizes the R package limma-voom [29, 30]. BCalm offers the same functionalities as mpralm, with additional features including preprocessing capabilities, the option to fit models to individual barcode counts rather than aggregated data, support for fitting sequences for comparisons within the same condition (referred to as element comparisons), and tools for analyzing element comparisons.

Mpralm estimates the logFC for each variant by fitting a linear model to the count data, employing voom to estimate the mean–variance relationship between the mean DNA count and the variance in log2 RNA/DNA ratios to account for overdispersion. While mpralm fits a linear model using the sum of barcodes in each technical replicate, BCalm enables fitting a linear model using each barcode count as a separate sample.

Mpralm has two methods for fitting a linear model, one designed for correlated input, e.g. for variants where reference and alternative allele belong to the same replicate, and one designed for independent input, e.g. for comparing between different cell types. Which column belongs to which replicate is described in a blocking vector, also used to normalize the counts per replicate. While mpralm requires this blocking vector only for fitting a correlated model, BCalm requires it for both models when modeling on barcode counts. For performing tests between conditions, a design matrix is required to indicate which count column belongs to which condition. BCalm provides a new wrapper function to fit data for a single condition, where no design matrix is required.

Specifically, the data is modelled using a generalized linear model (GLM) as follows:

$$\begin{array}{*{20}c} {Y = \beta _{0} + \beta X + \epsilon ,} & {\epsilon \sim N\left( {0,\sigma ^{2} W^{{ - 1}} \Sigma } \right)} \\ \end{array}$$

In the setting of variant effect estimation, the observations $Y{\text{ equal }}\log\frac{\text{RNA}_\text{alt}}{\text{DNA}_{\text{alt}}}$, the intercept $\beta_{0}{\text{ equals }}\log\frac{{RNA_{ref}}}{{DNA_{ref}}}$, and we have one coefficient $\beta_{1}$, which equals the logFC. The term $\varepsilon$ is an error term, which is assumed to be normally distributed. The design matrix, $X$, is 0 for each count belonging to a reference sequence and 1 for each count belonging to the alternative sequence. The precision weights $W$ are calculated using Voom, and ${\Sigma }$ is the covariance matrix capturing the correlation between counts observed within the same replicate. Limma estimates the residual variance $\sigma$, which is stabilized across all sequences using an empirical Bayes moderation method. By shrinking the variance of each tested sequence to a common value, limma controls the type I error.

In the setting of element activity estimation, the model is simplified to $Y = \beta_{0} + \epsilon$. The formulation of the counts as a GLM allows for flexibility in the test setup. One could, for instance, compare between different cell types by incorporating this in the design matrix.

To perform a statistical analysis between two groups within a single condition, typically between a test group and control group, we reimplemented limma’s TREAT [31]. TREAT performs a t-test relative to a specified threshold. The wrapper function accepts a percentile and a label for the negative control group. The percentile is used to calculate the logFC threshold from the negative control group, with a default value of 0.975, i.e. setting the upper threshold at the 97.5th percentile and the lower threshold at the 2.5th percentile of the logratio distribution of the negative controls. For performing the t-test, the distribution of the fitted logratios is shifted to be centered at the mean of the negative controls, which ensures correct testing when the upper threshold is negative. The method returns the fitted MArrayLM object, with the original unshifted logratios and the calculated p-values and t-statistics.

To provide users with better insight into a suitable threshold for the statistical test, BCalm includes plotting functionality that visualizes logratios for each group. The plotting function accepts an optional percentile argument, allowing the threshold to be calculated based on a specified negative control label and to be displayed alongside the logratios.

### Benchmarking methods

We benchmarked BCalm against mpralm and MPRAnalyze on the simulated dataset. Since MPRAnalyze does not scale to larger datasets (see Supplementary Table 1), only mpralm could be used with the lentiMPRA dataset. Mpralm used the aggregated barcode counts for each sequence. Both BCalm and mpralm are run with replicate count normalization, which is performed within the fitting routine.

As recommended by the authors, MPRAnalyze ran on data normalized using its estimateDepthFactors function with upper quantile normalization. Although MPRAnalyze is intended to model individual barcodes, we ran MPRAnalyze both on barcode counts and on aggregated counts for comparison. For running on individual barcodes, the replicate, allele and barcode information are used to fit the DNA model. For running on aggregated data, only the replicate and allele are used. Finally, we used MPRAnalyze’s analyzeComparative function for the simulated dataset.

## Results

### Different methods return similar logFCs but different statistics

**Fig. 1:**
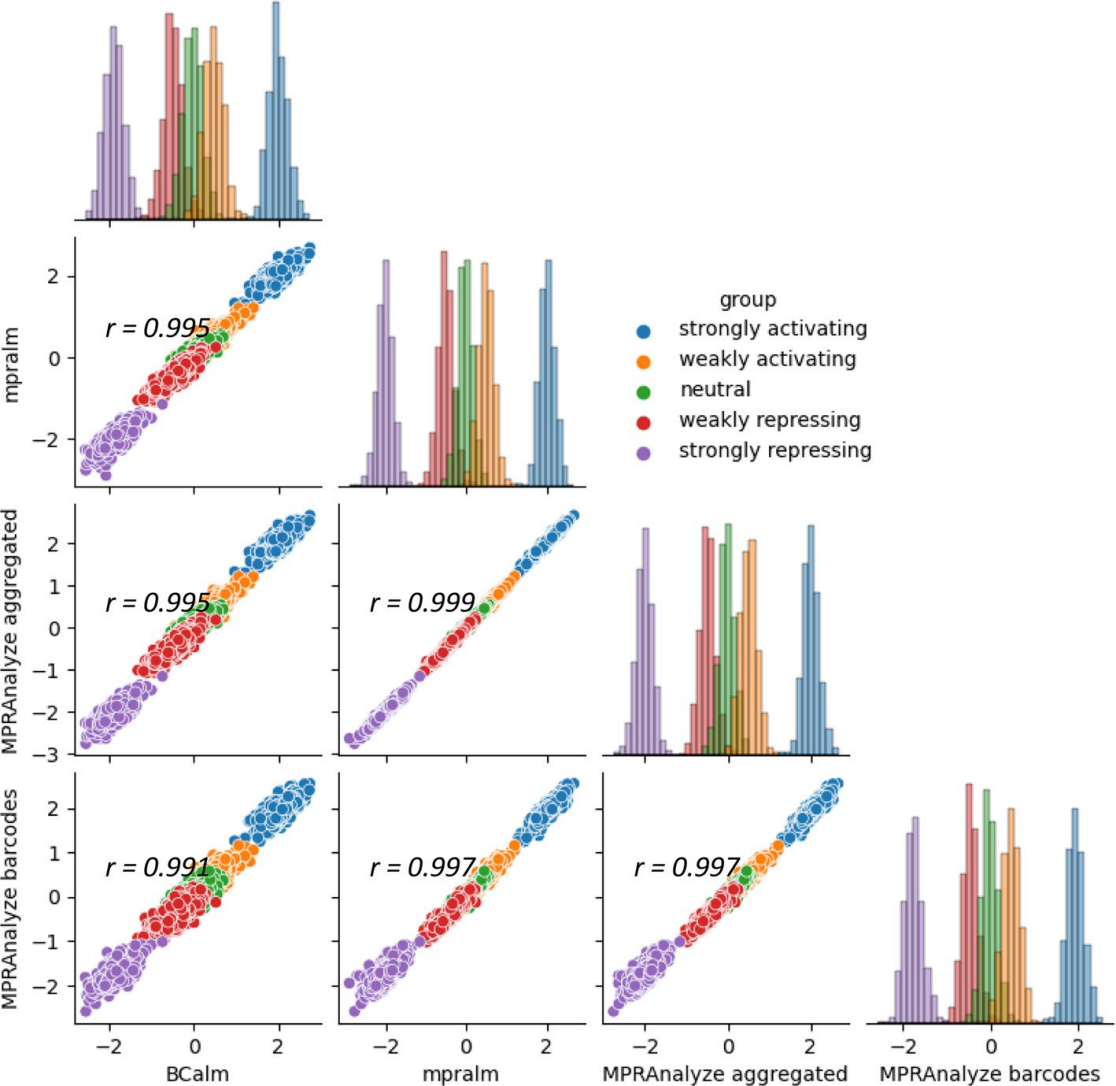
Scatter plots correlating logFCs estimated by BCalm, mpralm, MPRAnalyze “aggregated” and MPRAnalyze “barcodes” for simulated data, with Pearson correlations given in upper left corner. Count histograms are displayed on the diagonal. Data was simulated across five effect groups, highlighted with colors in the plots, as described in the methods

We ran BCalm, mpralm, a barcode-aggregated MPRAnalyze (custom) and barcode-level MPRAnalyze (default) on simulated variant datasets and correlated the resulting logFCs and p-values. The strongest correlation in estimated logFCs was observed between aggregated MPRAnalyze and mpralm, both of which use aggregated input (Fig. 1). Both aggregated methods were better at distinguishing between different effect groups based on logFCs when compared to the barcode methods. Despite these differences, the logFCs produced by all methods showed a generally very strong correlation (all greater than 0.99 Pearson correlation).

**Fig. 2:**
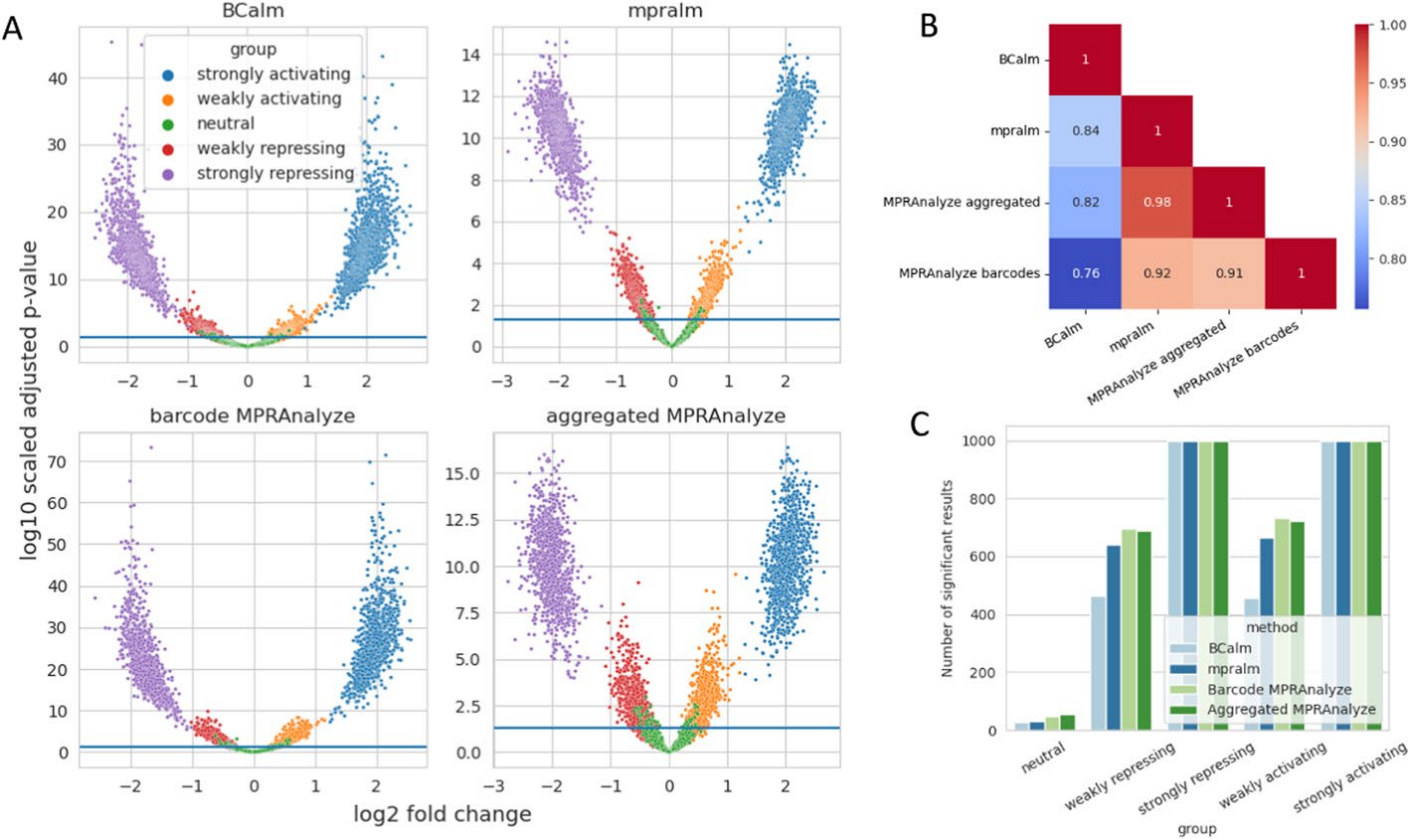
**A** Volcano plots showing the logFC on the x-axis and the BH-adjusted and log10 transformed p-values on the y-axis for each of the four methods on simulated data. Data was simulated across five groups as described in the methods and highlighted with colors in the plots. **B** Correlation between p-values calculated by different methods. **C** Number of significant results reported by each method for each of the five simulated groups described before. For the strongly activating and repressing groups, each 1000 significant results are expected, for the intermediate groups, 500 significant results are expected each. The neutral group should not result in significant results

After Benjamini-Hochberg (BH) correction, p-values show more distinct differences between the methods. As can be seen from the p-value range plotted on the y-axis in the volcano plots in Fig. 2A, the p-values for methods using barcode inputs get much smaller. The p-values of methods modeled on barcodes (BCalm and MPRAnalyze “barcodes”) correlate least with the p-values of aggregated methods, as can be seen in Fig. 2B. This is likely because the simulated stochastic variation between individual barcodes is averaged out when adding up barcode counts in the aggregation step. We investigate the p-value differences between MPRAnalyze and BCalm below.

BCalm returns the smallest number of significant results on the clean simulated dataset, while MPRAnalyze returns the highest number of significant results (see Fig. 2C). Each method returns all 2000 high effect variants as significant. Notably, BCalm has the smallest number of significant variants belonging to the neutral group.

### Limma-voom causes lower type I error than MPRAnalyze

**Fig. 3:**
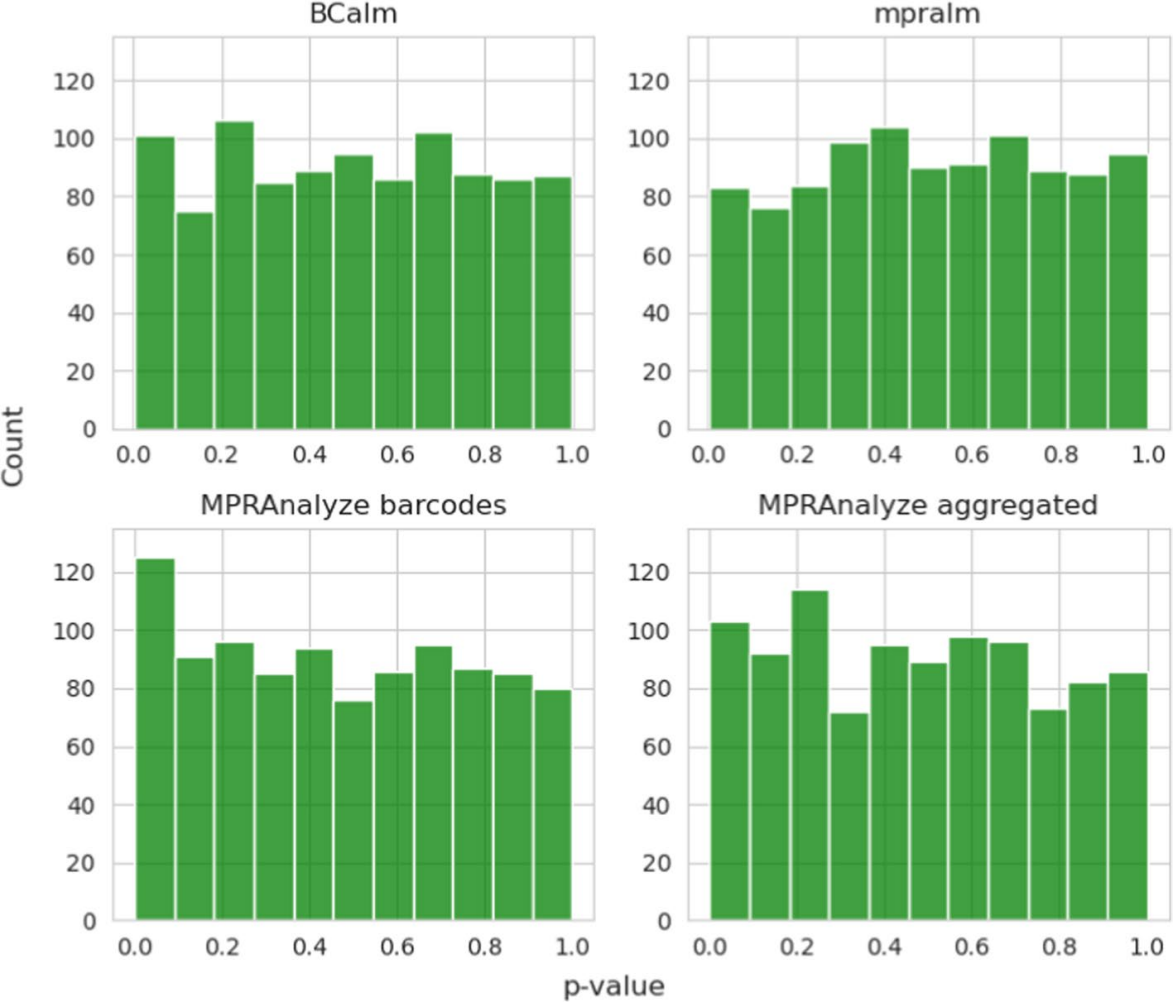
Distribution of p-values under the null hypothesis (i.e. the simulated neutral variant set) for all four benchmarked analysis methods. We expect a uniform distribution for all methods. Of the four plots shown, barcode-level MPRAnalzye shows the largest deviation from this expectation

We further examined the p-value distributions of the group with simulated neutral variants to investigate the behavior seen in Fig. 2B and C. In this group, effect sizes were simulated to follow a null distribution, meaning that the reference and alternative allele counts were drawn from the same distribution. Under these conditions, the p-values are expected to be uniformly distributed before adjusting for multiple testing [32]. We compared the p-values across all methods. As shown in Fig. 3, BCalm and mpralm exhibit the expected behavior, while the p-value distribution of MPRAnalyze is left-skewed towards zero, suggesting a higher type I error rate. While the left-skew is present for MPRAnalyze with and without barcode aggregation, it is most clearly seen for the default (barcode-level) analysis.

### Modeling on barcodes is more robust to outliers than modeling on aggregated data

**Fig. 4:**
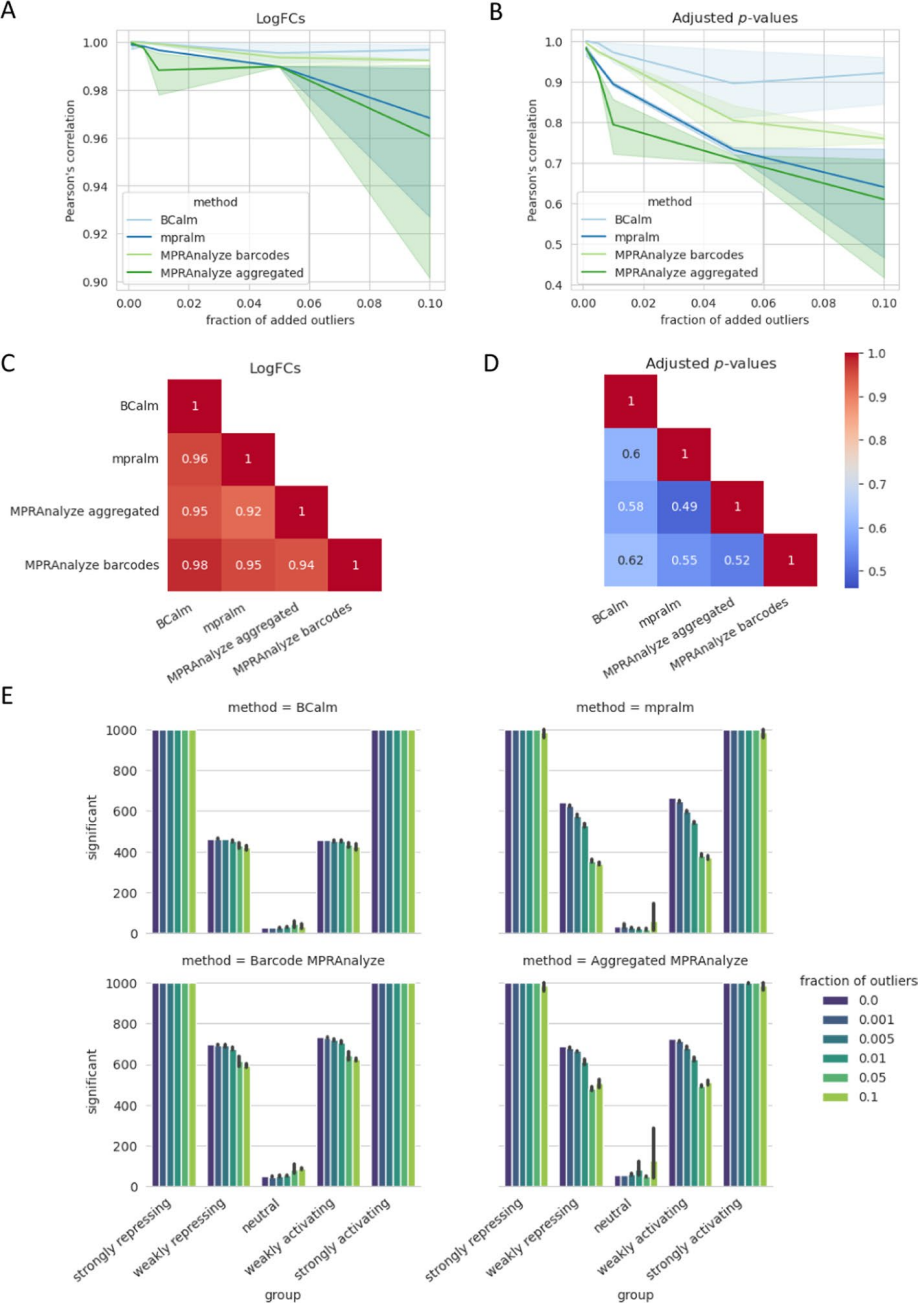
**A** Correlation of log fold changes and **B** adjusted p-values between clean simulated data and data with added outliers, repeated for five replications of random outlier additions. The four applied methods are shown as lines and value ranges observed for the random replicates are highlighted as colored areas. **C** Correlation between different methods of their reported logFCs and **D** adjusted p-values on simulated data at an outlier fraction of 0.1. **E** Number of significant variants for each of the four methods after outlier inclusion on simulated data. Error bars are given based on five replications of the random outlier additions. Each plot shows the five effect groups of simulated data as described in the methods. Bars are colored by the proportion of outliers added

Since real MPRA data is typically noisier than controlled simulated experiments, we additionally evaluated the performance of each method after introducing artificial outliers. We measured the Pearson correlation between the clean dataset and the dataset with outliers, as shown in Fig. 4A, and B. When barcode counts are aggregated, the correlation decreases more drastically compared to methods that model individual barcodes. For an outlier fraction of 0.1, BCalm correlates best with the other methods on both logFCs and adjusted p-values (see Fig. 4C, and D). As expected, BCalm correlates best with MPRAnalyze on barcode level, again suggesting advantages of barcode modeling when dealing with outliers.

Figure 4E illustrates the numbers of statistically significant variants returned by each method after adding outliers, which again reinforces that modeling on individual barcodes increases robustness to outliers. The aggregated approaches fail to detect all high-effect variants as significant and experience a higher reduction in the number of significant variants as the number of outliers increases. While MPRAnalyze consistently returns more significant variants than BCalm and mpralm, its number of false positives increases in the group of neutral variants. While BCalm returns the least significant variants on the clean dataset, its number of significant variants remains the most consistent after adding outliers. For outlier fractions 0.05 and 0.1, BCalm returns a higher number of significant variants than mpralm.

### BCalm is more robust to outliers in a lentiMPRA dataset

**Fig. 5:**
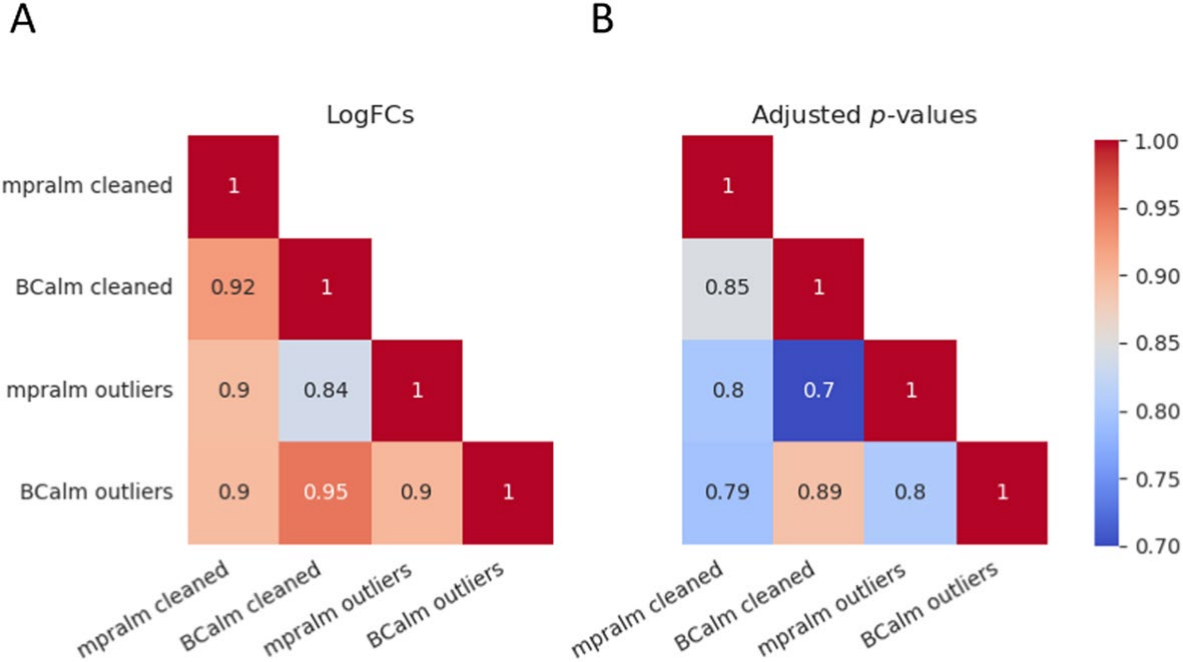
Correlation between logFCs (**A**) and adjusted p-values (**B**) reported by mpralm and BCalm on the lentiMPRA dataset with (“clean”) and without (“outliers”) removing outliers

We ran BCalm and mpralm on our library of 83,336 variants without removing outlier counts and 82,258 after removing outliers (as described in Methods). We observe that BCalm returns more consistent results. We correlated the logFC and adjusted p-values of the two methods (Fig. 5) and found that BCalm exhibits the highest correlation both with and without filtering outliers separately. Moreover, we found that mpralm correlates better with BCalm with outlier removal than with itself without filtering for outliers. These trends are consistent for both logFCs and adjusted p-values.

### BCalm finds more statistically significant variants in a lentiMPRA dataset

When using BCalm and mpralm to analyze the actual lentiMPRA variant library, BCalm finds more significant variants than mpralm, even after outliers are filtered separately (see Supplementary Table 1). Given that our simulations demonstrated that BCalm returns fewer significant results on a clean dataset (Fig. 4)), this discrepancy suggests that the lentiMPRA dataset may contain hidden outliers that are difficult to detect.

Both methods return a shared set of 2,493 significant variants, however, mpralm finds 796 additional variants that are not significant according to BCalm (“mpralm-only”) and BCalm finds 835 variants that are not significant according to mpralm (“BCalm-only”). To understand the consequences of missing some of these variants in the significant variant set, we tried to explore the biological relevance of the significant variants (see Table 1). Although the BCalm-only set has a total of 39 more variants, it has a higher proportion of variants located in an annotated transcription factor binding site (TFBS) or with CADD scores higher than 20 (i.e., the top 1% of predicted deleterious effects created by single nucleotide substitutions to the human reference genome), suggesting an enrichment for biologically meaningful effects. Specifically, 90 more variants in the BCalm-only set are located in a TFBS and 8 more are annotated to have a CADD score higher than 20.

**Table 1:** Absolute numbers of variants with possible biological relevance that are only reported using either BCalm or mpralm. As variants with possible biological relevance, we counted those located in an annotated transcription factor binding site (TFBS) by Ensembl Variant Effect Predictor and those annotated to have a Combined Annotation Dependent Depletion (CADD) score higher than 20 (i.e., that are among the top 1% of predicted deleterious effects created by single nucleotide substitutions to the human reference genome)

	Significant only with mpralm	Significant only with BCalm
Total	796	835
Located in TFBS	255 (32%)	345 (41%)
CADD score > 20	34 (4%)	42 (5%)

### BCalm is scalable to large datasets

We measured the runtime and memory used when running BCalm, mpralm, "aggregated" MPRAnalyze and "barcode" MPRAnalyze on the simulated dataset of 5000 variants (Supplementary Table 2). For all calculations, we used two threads on an Intel Xeon core in a SLURM high-performance compute (HPC) environment. Although modeling on individual barcodes increases runtime and memory (when using limma with BCalm and when using MPRAnalyze), the increase in runtime is more significant for MPRAnalyze. Even when MPRAnalyze uses aggregated input, it is slower than BCalm on individual barcodes. Since our lentiMPRA dataset is about 16 times larger than the simulated dataset, we decided to benchmark only BCalm and mpralm on the real dataset (Supplementary Table 2). As expected, the computational requirements of testing on a barcode-level (BCalm) are significantly higher, but with approximately 10 GB of memory and 10 min for 82,258 sequences still easily manageable with commonly available hardware.

### BCalm facilitates testing sequences relative to control sets

To accommodate the use of MPRAs to quantify activity of sequences relative to each other instead of another condition, BCalm uses limma’s TREAT function [31]. To validate and compare MPRA experimental outcomes, a tested library usually contains positive and negative controls which are expected to have activating, repressing, or no activity; selected according to literature and/or previous MPRA experiments. These control sequences can be used to find sequences with a statistically significant activity difference. To analyze such experiments, BCalm first calculates the logratios from the barcode counts. After this step, users can plot the activity for each labeled group based on the computed logratios (see Supplementary Fig. 1). When using a set of HepG2 specific negative controls to test for activating sequences relative to the 95th percentile of these negative controls, we find a total of 240 sequences that are significantly more active.

In this context, it is very important to understand the characteristics of the group of controls that is tested against and to include such description of tested elements with all experiments. Our group of HepG2 negative controls is comparatively neutral, i.e. its sequences still exhibit low activity and are for example not a set of repressing sequences (as can be seen from Supplementary Fig. 1). For this analysis, we can only draw conclusions about the activity of tested elements in relation to the control group, rather than in absolute terms.

## Discussion

We present BCalm, a package for testing element and variant effects from MPRA data. BCalm uses individual barcodes as an input to increase statistical power and outlier robustness, while staying scalable for large numbers of variants.

BCalm is designed to facilitate a seamless analysis of MPRA data for end users by providing code to preprocess outputs of MPRAsnakeflow, a workflow to convert raw sequencing data into count tables [24], and by offering plotting functionalities that aid in interpreting the final results.

We benchmarked BCalm against mpralm and MPRAnalyze using simulated data and observed strong correlations in the logFC values returned by each method. However, the choice of method becomes critical when performing statistical tests on MPRA data, as the p-values produced by different methods show weaker correlations and display distinct distributions. In a set of neutral variants, we demonstrated that MPRAnalyze exhibits an inflated type I error rate that is not observed for the other methods. While MPRAnalyze models the logFC per sequence individually and returns these values directly, limma performs an empirical Bayes variance moderation step. This step helps in controlling the type I error by shrinking the variance of all tests to a common value [33].

BCalm identified more significant results (and more biologically meaningful variants) in a real-world lentiMPRA dataset than mpralm. While experiments with simulated data indicated that aggregating input rather than modeling individual barcodes may yield more accurate results, this discrepancy likely arises from the additional noise introduced when considering each barcode individually. This additional noise from modeling barcodes individually is not problematic for real MPRA data, as the small variance between barcodes becomes negligible compared to the larger biological and technical noise present in actual experiments that deviate from the assumptions of a perfectly modeled negative binomial distribution. By comparing the results on a lentiMPRA dataset before and after removing outliers, we found that even after outlier removal BCalm returned more significant results. While we recommend always removing outliers before modeling MPRA counts, our experiments indicate again that real MPRA data contains noise that cannot be averaged out by aggregating barcode counts.

Testing based on individual barcodes results in a substantially larger input matrix than for aggregated counts, which can be problematic when memory is limited. Generally, both runtime and memory use increase when modeling on individual barcodes (see Supplementary Table 1). Still, BCalm can analyze the same dataset of 5000 variants in less than two minutes, where MPRAnalyze requires nine minutes on aggregated input and 488 min on barcodes. For situations where modeling individual barcodes is impractical, BCalm supports using aggregated input.

Finally, we showed BCalm’s functionality for testing sequences relative to a set of negative controls on our lentiMPRA dataset using limma TREAT [31]. For a detailed discussion on the benefits of using TREAT over a logFC cutoff or a traditional t-test, please refer to its original manuscript.

## Conclusion

MPRAs are a useful resource for assessing expression effects of regulatory sequences and measuring the impact of their sequence perturbations. While they have seen broader adoption by the community over the past years, there are many variables that cause noise and outliers in the count data that can be hard to detect. We present BCalm, a method that models the activity of sequences on individual barcode counts to increase robustness to outliers. By modeling barcode-level counts instead of technical replicates only, the sample size and therefore the statistical power of the model increases, which can be especially useful when few technical replicates are available.

We compared this approach to MPRAnalyze, another method that models individual barcodes, and mpralm, a method that builds upon the limma-voom framework. We found that MPRAnalyze is affected by a higher type I error compared to BCalm and mpralm. BCalm has increased statistical power, is scalable to large MPRA libraries and more robust to outliers than mpralm. We provide routines to directly use files from MPRAsnakeflow, offer plotting functionalities and enable the testing of sequences relative to a group of negative controls. With BCalm, we provide a new tool to the community that simplifies the transition from MPRA count tables to analysis.

## Supplementary Information

Additional file 1.

## Data Availability

The lentiviral MPRA dataset is publicly accessible at the IGVF portal https://data.igvf.org using accession code IGVFSM9009DVDG. The package is accessible at https://github.com/kircherlab/BCalm, while https://github.com/kircherlab/BCalm_experiments contains the code for the validation experiments along with a file for the simulated dataset.
