# Supplementary material for: Using individual barcodes to increase quantification power of massively parallel reporter assays
Source: BMC Bioinformatics. 2025 Feb 13;26:52. doi: 10.1186/s12859-025-06065-9 (PMC11827149; doi:10.1186/s12859-025-06065-9)
Supplement: Supplementary file 1 — Additional file 1. [file 12859_2025_6065_MOESM1_ESM.docx]

Supplementary Materials: Using individual barcodes to increase quantification power of Massively Parallel Reporter Assays

Pia Keukeleire, Jonathan D. Rosen, Angelina Göbel-Knapp, Kilian Salomon, Max Schubach and Martin Kircher

**Supplementary Figure 1**


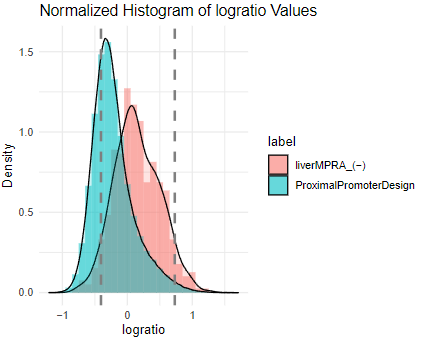


Supplementary Figure 1: BCalm plotting functionality shows logratios of tested and negative control groups, as well as a threshold at a user defined percentile of the negative controls (default: 0.95).

**Supplementary Table 1:** Analyzing the lentiMPRA variant library with BCalm and mpralm with and without separate barcode outlier removal. While the number of significant variants increases considerably for mpralm when outliers are filtered separately, BCalm finds more significant variants in both settings.

| Method | Outlier removal | Number of significant variants |
| --- | --- | --- |
| Mpralm | Yes | 2780 |
| BCalm | Yes | 3856 |
| Mpralm | No | 3289 |
| BCalm | No | 3328 |

**Supplementary Table 2**: Computational requirements as measured by runtime for each method on a barcode input of 5,000 simulated variants. Resources were measured using R and SLURM’s “seff” command using 2 cores in a SLURM HPC environment.

| Data set | Method | Runtime | Memory |
| --- | --- | --- | --- |
| Simulated | MPRAnalyze aggregated | 9.03 minutes | 897MB |
| Simulated | MPRAnalyze barcodes | 487.59 minutes | 1.76GB |
| Simulated | Mpralm | 0.07 minutes | 96KB |
| Simulated | BCalm | 1.47 minutes | 1.85GB |
| HepG2 lentiMPRA | Mpralm | 1.29 minutes | 5.7GB |
| HepG2 lentiMPRA | BCalm | 10.33 minutes | 9.7GB |

**Supplementary Table 3:** Comparison of the methods using the properties statistical power, robustness to outlier, speed, Type I error (i.e. false positives), Type II error (i.e. false negatives), and the statistical model used to fit the data.

|  | MPRAnalyze | mpralm | BCalm |
| --- | --- | --- | --- |
| statistical power | high | middle | high |
| robustness to outlier | high | low | high |
| speed | low | high | high |
| Type I error | middle | low | low |
| Type II error | low | middle | low |
| statistical model | negative binomial distribution | limma-voom | limma-voom |
